# Immune Response to Third and Fourth COVID-19 Vaccination in Hemodialysis Patients and Kidney Transplant Recipients

**DOI:** 10.3390/v14122646

**Published:** 2022-11-26

**Authors:** Patrick Affeldt, Felix Carlo Koehler, Karl August Brensing, Martin Gies, Eva Platen, Vivien Adam, Linus Butt, Franziska Grundmann, Eva Heger, Steffen Hinrichs, Nils Kalisch, Simon Oehm, Gertrud Steger, Maike Wirtz, Thomas Benzing, Dirk Stippel, Florian Klein, Christine Kurschat, Roman-Ulrich Müller, Veronica Di Cristanziano

**Affiliations:** 1 Department II of Internal Medicine and Center for Molecular Medicine Cologne, Faculty of Medicine and University Hospital Cologne, University of Cologne, 50937 Cologne, Germany; 2 Institute of Virology, Faculty of Medicine and University Hospital of Cologne, University of Cologne, 50935 Cologne, Germany; 3 CECAD, Faculty of Medicine and University Hospital of Cologne, University of Cologne, 50931 Cologne, Germany; 4 Nierenzentrum Bonn, 53175 Bonn, Germany; 5 KfH-Nierenzentrum Köln-Longerich, 50737 Cologne, Germany; 6 Nierenzentrum Eifel, 53894 Mechernich, Germany; 7 Department of General, Visceral, Cancer and Transplant Surgery, Faculty of Medicine and University Hospital Cologne, University of Cologne, 50937 Cologne, Germany

**Keywords:** BA.1, dialysis, immunosuppression, neutralization, protection, surrogate, kidney disease, SARS-CoV-2, transplantation, booster

## Abstract

Severe acute respiratory syndrome coronavirus type 2 (SARS-CoV-2) is a serious hazard for hemodialysis (HD) patients and kidney transplant (KTX) recipients as they suffer from an impaired immune response to SARS-CoV-2 vaccination. In addition, a definition of SARS-CoV-2 IgG titer that indicates a sufficient immune response, especially against new omicron variants, is urgently needed. In the present study, the immune response to either a third or a fourth dose of a mRNA vaccine was investigated in 309 dialysis and 36 KTX patients. SARS-CoV-2 IgG titer thresholds indicating neutralizing activity against wild type (WT) and the omicron variant BA.1 were quantified. After four vaccine doses, a high-neutralizing activity against WT was evidenced in HD patients, whereas the neutralizing rate against BA.1 was significant lower. Concerning KTX recipients, humoral and cellular immune responses after a third vaccination were still highly impaired. This calls for modified omicron-targeting vaccines.

## 1. Introduction

As hemodialysis (HD) patients and kidney transplant (KTX) recipients suffer from an impaired immune system, they are at risk for severe coronavirus disease-19 (COVID-19) [1]. Apart from seroconversion, longevity of immune response to severe acute respiratory syndrome coronavirus type 2 (SARS-CoV-2) after infection and vaccination is reported to be disturbed in both patient cohorts [2,3,4,5,6,7]. Age, frailty and severity of pre-existing comorbidities are prognostic factors for severe COVID-19 in both immunocompromised and immunocompetent patients [8]. Chronic kidney disease (CKD), HD or a pre-existing solid organ transplantation are linked to higher mortality upon SARS-CoV-2 infection [9,10,11]. In addition, risk of infection is increased in HD patients due to challenges in social distancing in dialysis centers and during the travel to and from the centers [3,12,13,14].

Currently, active immunization against SARS-CoV-2 is considered the most effective prevention strategy for vulnerable individuals from both infection and severe disease [3]. In particular, the messenger ribonucleic acid (mRNA) vaccines mRNA-1273 (Moderna, CA, USA) and BNT162b2 (BioNTech/Pfizer, Mainz, Germany/New York City, NY, USA) induce an effective antiviral humoral and cellular immune response [15,16]. Both contain lipid nanoparticle-encapsulated, nucleoside-modified mRNA that encodes the SARS-CoV-2 spike protein. mRNA-1273 is administered in doses of 100 μg 28 days apart, whereas BNT162b2 vaccine is administered as a 30 μg dose 21 days apart [15,16].

However, HD patients and especially KTX recipients show a diminished immune response to the vaccine with reduced seroconversion rates, lower antibody titers and a delayed immune response [1,3,17,18]. Notwithstanding, after the second mRNA vaccine dose, seroconversion rates may be as high as 70–96% and two doses of mRNA-1273 induce higher antibody titers than two doses of BNT162b2 in HD patients [3,19,20]. In contrast to this, seroconversion rate following two doses of mRNA vaccines in KTX can be as low as 50% and a third dose induces a serologic response in approximately 50% of patients not responding after two doses [3,7,21].

As vaccine-induced immunogenicity is reported to vanish rapidly, the administration of booster doses is recommended for vulnerable patients, including HD and KTX patients [22]. In addition, with the spread of the immune-escaping omicron variant, a booster dose of mRNA vaccine is considered critical to generate an effective neutralizing response against this variant [23,24,25]. However, intensity and duration of the immune response upon third and fourth mRNA vaccine doses in these groups of patients are still unclear and the definition of protection conferring antibody titers, especially against the omicron variant, remains unaddressed [26]. Furthermore, the difference in vaccine-induced immunogenicity in the case of mRNA SARS-CoV-2 vaccines is not well characterized. Therefore, we retrospectively analyzed seroconversion rate and antibody levels targeting the SARS-CoV-2 spike protein and its receptor binding domain (RBD) after third and fourth vaccination in 309 HD patients vaccinated with BNT162b2 or mRNA-1273 and prospectively analyzed the humoral and cellular immune response in 36 KTX patients.

## 2. Materials and Methods

### 2.1. Study Design, Patients, and Ethical Statement

We retrospectively analyzed anti-SARS-CoV-2 antibody levels in 309 HD patients after three full doses of an mRNA vaccine. In addition, anti-RBD IgG titers after four vaccine doses were available for 182 HD patients (Table 1).

The mRNA-1273 was administered with a dose of 100 μg, as currently recommended [26]. Patients with known SARS-CoV-2 infection after booster-immunization were excluded from this study. The third vaccination was administered in the Cologne–Bonn region (Western Germany) between September and December 2021 (prevalent circulating variant Delta) and the fourth vaccination in April 2022 (prevalent circulating variant BA.2). The results were retrospectively analyzed as approved by the local institutional review board (Ethics Committee of the Medical Faculty of the University of Cologne, Germany, EK 21-1398) in line with the Declaration of Helsinki.

Regarding KTX patients, humoral and cellular immune responses after the administration of various vaccination schemes were available for 36 patients. Laboratory measurements were performed prospectively after approval from the local institutional review board (Ethics Committee of the Medical Faculty of the University of Cologne, Germany, EK 21-1112) in line with the Declaration of Helsinki.

### 2.2. Serological Assays Detecting Anti-SARS-CoV-2 Antibodies

IgG targeting the receptor binding domain (RBD) of the spike protein were quantified by the chemiluminescent microparticle immunoassay (CMIA) SARS-CoV-2 IgG II Quant by Abbott on the automated system Alinity I (Abbott, Abbott Park, IL, USA). In addition, the quantitative determination of antibodies targeting additional regions of the spike protein was performed using the chemiluminescence immunoassay (CLIA) LIAISON^®^ SARS-CoV-2 TrimericS IgG assay by DiaSorin on the platform Liaison XL (Diasorin, Vicenza, Italy), which measures IgG against a recombinant trimeric spike antigen, including S1 and S2, as well as the Euroimmun anti-SARS-CoV-2-QuantiVac-ELISA (Enzyme-linked Immunosorbent Assay) on the Euroimmun Anlalyzer I (Euroimmun Diagnostik, Lübeck, Germany) that quantifies IgG against the S1 region. All anti-SARS-CoV-2 IgG titers were expressed in binding antibody units per milliliter (BAU/mL), according to the conversion factor provided by each manufacturer based on the World Health Organization (WHO) international standard. All assays were performed and interpreted according to manufacturer’s recommendations.

### 2.3. Live Virus Assay to Determine Serum Neutralizing Activity against SARS-CoV-2 Wild Type (WT) and Omicron Variant BA.1

The neutralizing serum activity against SARS-CoV-2 wild type (WT) was performed as previously described [27]. In the case of live virus neutralization assay against BA.1, the virus was isolated from a nasal swab using VeroE6 cells and then expanded in culture by superinfection of VeroE6 from the initial outgrowth culture. Whole genome sequencing of isolated virus was performed through Illumina sequencing. Serum samples were serially diluted (1:10, 1:50, 1:250, 1:1250, 1:6250, 1:31,250) and mixed with 100 TCID50 (50% tissue culture infectious dose) of live virus. The virus–serum mixture was incubated for one hour at 37 °C. Afterwards, 50 μL of VeroE6 cell suspension (250,000 cells/mL) were added to each sample dilution. Cells were incubated at 37 °C for 4 days before determining virus-related cytopathic effects (CPE) microscopically, as previously described [27].

### 2.4. Determination of T Cell Response to SARS-CoV-2

The CD4+ and CD8+ T cell-mediated immune responses to SARS-CoV-2 were measured by the commercial whole blood interferon-γ (IFN-γ) release assay (IGRA) QuantiFERON SARS-CoV-2 (QIAGEN GmbH, Hilden, Germany). T cells were stimulated with epitopes of S1 and S2 subunits of SARS-CoV-2 spike protein (antigen 1 and 2). IFN-γ concentration was quantified by ELISA. According to manufacturer’s recommendations, an IFN-γ level ≥0.15 IU/mL was interpreted as reactive.

### 2.5. Statistics

For statistical analyses we used GraphPad Prism software version 5 (GraphPad Prism Software Inc. San Diego, CA, USA) and SPSS version 27.0 (SPSS Inc., Chicago, IL, USA). We summarized non-normal distributions of antibody titers as median ± interquartile range and compared them using the Kruskal–Wallis/Mann–Whitney *U* tests. Regarding patient characteristics, numerical variables were expressed by median and interquartile range and categorical variables as absolute numbers and frequencies, respectively. Numerical variables were compared using the Kruskal–Wallis/Mann–Whitney *U* test, whereas categorical variables were analyzed using the chi square test in a descriptive manner.

Comparisons between differences in the subgroups reaching the cut-offs for omicron and WT were performed using the chi square tests. For adjusting differences in patients reaching the cut-offs to different factors of influence (age, sex, years of dialysis therapy, infection with SARS-CoV-2 prior to booster-immunization, days between basic- and booster immunization and days between vaccination and blood sampling), a binary regression model was performed.

A receiver operating characteristic (ROC) analysis was performed to determine levels of SARS-CoV-2 IgG with sufficient neutralizing activity against WT and BA.1. A passing Bablok regression model (Analyze-it v5.50, Analyse-it Software, Ltd. Leeds, UK) was employed to determine whether the results obtained by commercial serological assays targeting different regions of SARS-CoV-2 were comparable.

## 3. Results

### 3.1. SARS-CoV-2 Anti-RBD IgG Titers in Hemodialysis Patients after Third and Fourth Vaccination

**Figure 1:**
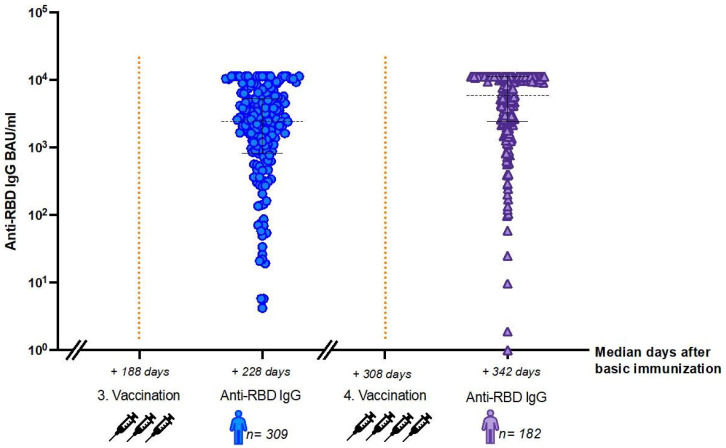
SARS-CoV-2 anti-receptor binding domain (RBD) IgG responses after third (blue) and fourth (purple) vaccination. Median days after the second dose are given on the x-axis and anti-RBD IgG titers on the y-axis. Doted black lines indicate median SARS-CoV-2 IgG titers and solid black lines correspond to the interquartile range (IQR).

Anti-RBD SARS-CoV-2 IgG titers were available after third and fourth vaccination for 309 and 182 HD patients, respectively. Baseline characteristics are summarized in Table 1. Patients received a third and fourth vaccination at a median of 188 days and 308 days after the second dose, respectively. IgG titers were measured after a median of 40 days after receiving the third dose and 34 days after the fourth one (Table 1 and Figure 1).

The median SARS-CoV-2 anti-RBD IgG titer in HD patients after four vaccine doses was 6923.5 BAU/mL (IQR 8661.15 BAU/mL) and thus significantly higher in comparison to the IgG titer after the third vaccination (2882.70 BAU/mL) (IQR 3093.00 BAU/mL; *p* < 0.001) (Figure 1).

**Figure 2:**
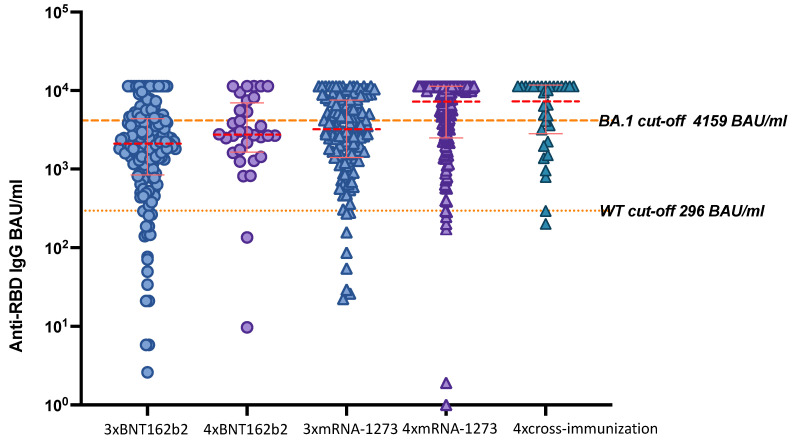
Anti-receptor binding domain (RBD) IgG response after three and four doses of an mRNA vaccine in hemodialysis (HD) patients. Doted red lines indicate median SARS-CoV-2 IgG titers and solid red lines correspond to the interquartile range (IQR). Doted orange lines represent examined cut-offs for neutralizing activity against WT and BA.1.

Of note, HD patients receiving four doses of the mRNA-1273 vaccine revealed higher anti-RBD IgG levels than patients vaccinated with four doses of BNT162b2 (*p* < 0.001) (Table 2). The highest antibody titers among HD individuals were induced by cross vaccination using both mRNA-1273 and BNT162b2 (Kruskal–Wallis test, *p* = 0.002) (Figure 2).

### 3.2. Comparison of Anti-Spike IgG Titers Quantified with Different Commercial Serological Assays

**Figure 3:**
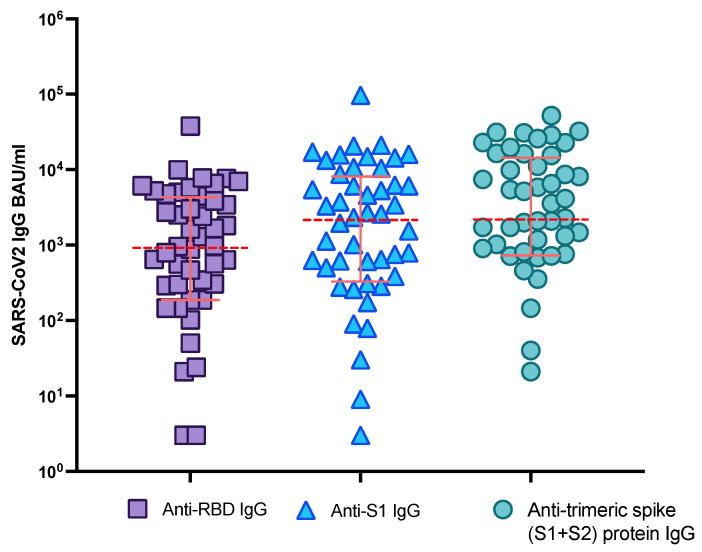
Comparison between commercial serological assays targeting different regions of the SARS-CoV-2 spike protein. Doted red lines indicate median SARS-CoV-2 IgG titers and solid red lines correspond to the interquartile range (IQR).

Anti-RBD, anti-S1, and anti-trimeric spike protein IgG titers as well as the neutralizing serum activity against WT and BA.1 were available for 48 HD patients vaccinated with four doses of either mRNA vaccine (Figure 3, Supplemental Figure S1).

Multiple passing Bablock regression models showed that SARS-CoV-2 IgG titers quantified by different tests—Abbott (anti-RBD IgG, CMIA), Euroimmun (anti-S1 IgG, ELISA) and Diasorin (anti-trimeric spike protein IgG, CLIA)—were difficult to compare, even after conversion into BAU/mL according to the WHO international standard (Supplemental Figure S1).

### 3.3. Determination of IgG Titers as a Surrogate Marker of the Neutralizing Activity against SARS-CoV-2 after the Fourth Vaccination in Hemodialysis Patients

**Figure 4:**
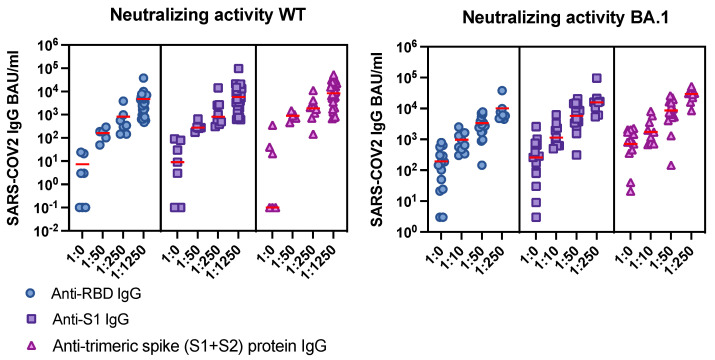
Correlation between SARS-CoV-2 IgG titers and the serum neutralizing activity against wild type (WT) and the omicron variant BA.1. Solid red line: median titer.

As mentioned above, IgG titers measured by commercial assays targeting different regions of the spike protein were not directly comparable, even after adjusting for BAU/mL. To better elucidate these differences, the neutralizing activity against WT and BA.1 for specific levels of SARS-CoV-2 IgG after the fourth vaccination was investigated using multiple ROC analyses in a cohort of 48 HD patients (Figure 4). In the case of anti-RBD IgG, the ROC analysis revealed a titer of ≥296 BAU/mL (WT cut-off) to have the highest sensitivity (0.94) and specificity (1.0) for predicting a meaningful serum neutralizing activity against the WT (≥1:250) (Figure 4, Supplemental Table S1, Supplemental Figure S2).

Titers of anti-trimeric spike IgG ≥ 1100 IgG BAU/mL (sensitivity: 0.85, specificity: 0.92) and of anti-S1 protein IgG ≥ 298 BAU/mL (sensitivity: 1.0, specificity: 0.92) were identified to reach a comparable serum neutralizing activity (≥1:250) against WT (Figure 4, Supplemental Figure S4, Supplemental Table S3).

Concerning omicron, our ROC analysis revealed an anti-RBD IgG titer of ≥4159 BAU/mL (omicron cut-off) to have the highest sensitivity (1.00) and specificity (0.90) predicting a serum neutralizing activity against BA.1 (≥1:10) (Figure 4, Supplemental Table S2, Supplemental Figure S3). In addition, titers of anti-trimeric spike protein IgG ≥ 1850 BAU/mL (sensitivity: 0.77, specificity: 0.93) and of anti-S1 IgG ≥ 773 BAU/mL (sensitivity: 0.87, specificity: 0.93) were found to predict a serum neutralizing activity against BA.1 (≥1:10) (Figure 4, Supplemental Figure S5, Supplemental Table S4).

### 3.4. Anti-RBD-IgG Titers with Potential Neutralizing Activity against WT in Dialysis Patients

As previously described, a level of ≥296 BAU/mL anti-RBD-IgG was found to elicit meaningful neutralizing activity against WT (≥1:250). After three doses of any mRNA vaccine, 272 out of 309 dialysis patients (88.03%) had a titer ≥296 BAU/mL (Table 3). Comparing IgG titers after three doses of mRNA-1273 or BNT162b2, the number of patients not reaching a titer of ≥296 BAU/mL was low and did not differ between both groups (mRNA-1273: n = 9, BNT162b2: n = 18, *p* = 0.21) (Figure 2, Supplemental Table S5). After four doses of an mRNA vaccine, only 9 out of 182 (5.0%) HD patients did not reach the threshold for WT (Table 3). Again, the number of patients not reaching the WT cut-off of ≥296 BAU/mL did not differ between mRNA-1273 (5 out of 118) and BNT162b2 (2 out of 32) (*p* = 0.641) (Supplemental Table S7).

### 3.5. Anti-RBD-IgG Titers with Potential Neutralizing Activity against Omicron Variant BA.1 in Dialysis Patients

We determined a cut-off of ≥4159 BAU/mL anti-RBD-IgG to predict neutralizing activity against BA.1 at all (≥1:10) (omicron cut-off). However, only 30.8% percent of HD patients having received three doses of an anti-SARS-CoV-2 vaccine, regardless of the underlying vaccine scheme, achieved this cut-off (Table 3). Of note, a significantly higher number of HD patients vaccinated with mRNA-1273 had IgG titers eliciting the BA.1 cut-off than those with BNT162b2 (55/130 vs. 40/179, *p*= 0.002) (Supplemental Table S6). This difference was still significant in a binary regression model adjusting for age, sex, years of dialysis therapy, infection with SARS-CoV-2 prior to booster-immunization, days between basic and booster immunization, and days between vaccination and blood sampling (*p* = 0.001) (Supplemental Table S9).

The BA.1 cut-off was achieved in 64.3% of HD patients having received four vaccine doses (Table 3). After three doses of a mRNA vaccine, the proportion reaching detectable neutralizing titers against BA.1 was significantly higher in HD vaccinated with mRNA-1273 than those with BNT162b2 (86/118 vs. 10/32, *p* > 0.001) (Supplemental Table S8). However, the between-group differences did not reach significance anymore after being adjusted for age, sex, years of dialysis therapy, days between basic- and booster immunization (*p* = 0.095) (Supplemental Table S10).

### 3.6. Humoral and Cellular Immune Responses after SARS-CoV-2 Vaccination in Kidney Transplant (KTX) Recipients

Prospective monitoring for humoral and cellular immunity was available for a total of 36 KTX patients (32 after second vaccination, 29 after third vaccination; Table 4). Although heterogeneous vaccination schemes were used, only 10 out of 29 KTX patients had a measurable humoral immune response after three doses (median anti-RBD IgG 134.4 BAU/mL (IQR 218.4), Table 5). In addition, only in two of those patients a low neutralizing activity against WT could be measured (Supplemental Table S11). Specific T cell reactivity against SARS-CoV-2 was detected in two patients only (Table 5, Supplemental Table S11).

## 4. Discussion

KTX and dialysis therapy are known risk factors for a diminished immune response to SARS-CoV-2, with reduced seroconversion rates, lower antibody titers and a delayed immune response. So far, there is no uniform definition of protection conferring antibody titers, especially against the omicron variant, that—with its heterogenous number of subtypes—is currently the predominant variant of concern (VOC) worldwide [1,3,17,18,23,24,25]. Booster doses of mRNA vaccines are considered critical to generate an effective neutralizing response against immune escape variants [23,24,25].

In the analysis at-hand, we investigated the humoral response following a third and fourth dose of anti-SARS-CoV-2 mRNA vaccines in HD patients. Kidney failure is a known risk factor for insufficient and waning immunity to vaccines [28]. In line with previous reports, we detected that mRNA-1273 elicited higher antibody levels compared to BNT162b2 [3,19,20]. After booster vaccinations, the overall rate of non-responders against SARS-CoV-2 WT was very low and a fourth dose induced a significant increase in antibody titers [29,30,31]. Interestingly, we found that cross immunization schemes employing both mRNA vaccines induced the highest antibody titers.

The determination of neutralizing serum activity represents the most reliable laboratory method to determine the level of antibody protection from infection and severe disease [32]. However, the measurement of neutralizing response has not yet been established in clinical routine diagnostics. Of note, there is a high correlation between anti-spike IgG concentration and the level of neutralization [33]. Therefore, determination of SARS-CoV-2 IgG titers as a surrogate marker of protection from infection and disease is pending.

IgG titers targeting the spike protein are reported in BAU/mL, according to the WHO international standard. In line with recent findings, antibody titers measured using serological assays targeting different S protein antigens were not one-to-one comparable in our analysis, indicating the need for differentiated interpretation of measured titers depending on the assay [34]. To find out which antibody titers targeting different regions of the spike protein (anti-trimeric spike protein, S1, or RBD) were specifically associated with neutralizing serum activity, we performed a ROC analysis in HD patients receiving four doses of a mRNA vaccine. A neutralization titer of ≥1:250 assessed in live virus neutralization assays was considered a valid cut-off for protection against SARS-CoV-2 WT [35]. For anti-RBD IgG, a cut-off of 296 BAU/mL was the most appropriate titer to predict an effective neutralizing activity against WT virus, in line with recent findings [36,37]. A similar cut-off was found when considering anti-S1 IgG (whole S1 compared to RBD), whereas a higher titer as a cut-off of protection was identified in the case of anti-trimeric spike protein-based serological assays. Regarding BA.1, a much higher anti-RBD titer of 4159 BAU/mL was suitable to predict the evidence of neutralizing activity at all (≥1:10), reflecting the omicron’s high mutation rate in the RBD of the spike protein [38]. In the case of omicron, the difference between the antibody cut-offs for neutralization measured by different serological assays became more marked. Thus, cut-offs for antibody titers as a surrogate maker of protection against SARS-CoV-2 should be defined for every region of spike protein-targeting antibodies.

We determined that 88% of HD patients reached the WT cut-off for neutralization after three and more than 90% after four vaccine doses. In contrast, titers conferring neutralizing activity against BA.1 were detected in only 30% of patients after three doses and in 64% following four doses. Therefore, upcoming omicron-modified vaccines are urgently needed, especially for patients at higher risk for severe disease [26,39]. Moreover, in the case of SARS-CoV-2 infection, pre-emptive therapeutic intervention, including passive immunization and antiviral drugs, should be considered for high-risk patients, regardless of their current vaccination status [9,26].

In line with previous reports, mRNA-1273 vaccine induced significantly higher antibody titers compared to vaccination with BNT162b2 [3,19,20]. However, less is known about the differences in neutralizing activity between the two mRNA vaccines in HD patients. In our study, more patients reached the omicron cut-off for neutralization following three doses of mRNA-1273 compared to BNT162b2. After four doses of an mRNA vaccine and adjusting for age, sex, years of dialysis therapy, days between basic and booster immunization, there was a strong trend that failed to reach significance of a higher BA.1 neutralization activity after mRNA-1273 vaccination. Nonetheless, any mRNA vaccine should be used according to local availability and potential differences in side effect profiles may also have to be taken into account.

KTX patients are considered at-risk for severe disease due to their immunosuppressive therapy. Since the beginning of the SARS-CoV-2 pandemic, the management of infected immunosuppressed patients has largely improved. However, hospitalization and mortality reported in this group of patients remain significantly high compared to the general population [40]. In a previous study, we reported a weak immune response following two vaccine doses in KTX recipients [3]. In the study at-hand, humoral and cellular immunity were again prospectively monitored after third vaccination in a group of 36 KTX recipients. In general, seroconversion was observed in just ten patients with all patients having antibody titers below the determined cut-off of antibody protection of 296 BAU/mL. Three patients were tested reactive by SARS-CoV-2 QuantiFERON. In contrast to previous data, the immune response induced by the vaccine was lower than expected in our cohort. Available data reported that a third dose of mRNA vaccine elicited an antibody response in half of renal transplant patients who failed to respond to the first two doses [7,41]. Osmanodja et al. (2022) showed that repeat vaccinations, up to five doses, induced a humoral response in up to 90% of KTX recipients [42]. As immunosuppressive therapy is a known risk factor for lacking seroconversion after vaccination, it is important to note that our cohort was examined earlier after TX and had strong immunosuppression compared to other findings in the field. In addition, most patients received immunosuppressive therapy containing MPA or MMF, which is a known risk factor for lacking seroconversion [3]. Recent data indicate that temporary halting of antimetabolite treatment may increase the seroconversion rate in KTX patients [43].

Our study has some limitations. As data from HD patients were derived from routine laboratory samples, patients were heterogeneous with regard to time intervals of vaccination and serological testing. Additionally, since data were retrospectively collected and analyzed, we had no influence on the choice of vaccines in HD patients. For this reason, vaccine schemes were heterologous and not equally distributed in this cohort.

Strengths of our study include the large number of analyzed patients, the determination and comparison of vaccine titers against different regions of spike proteins, and the definition of antibody cut-offs as a surrogate marker of neutralization against both the WT virus and the omicron variant.

## 5. Conclusions

Booster vaccinations for vulnerable groups represent an important strategy to overcome the impairment of the immune system especially regarding the emerging SARS-CoV-2 variants [44,45]. Routine serological testing in KTX patients to assess the level of immune response after vaccination could contribute to identifying patients with an inadequate humoral response, based on the identified antibody cut-offs as a surrogate marker of virus neutralization. These patients are candidates for further booster vaccinations.

## Figures and Tables

**Table 1:** Baseline characteristics of hemodialysis (HD) patients receiving third and fourth anti-SARS-CoV-2 vaccination. Vaccine used: mRNA-1273 (Moderna, CA, USA) and BNT162b2 (BioNTech/Pfizer, Mainz, Germany/New York City, NY, USA).

**Hemodialysis (HD) Patients after Third Vaccination**	**All HD** **Patients** **N = 309** **(100%)**	**3xBNT162b2 N = 179** **(48.8%)**	**3xmRNA1273** **N = 130 (35.4%)**	
Age in years, median (IQR)	68 (23.75)	68 (24)	66 (23,5)	
Sex, N (%)				
Female	119 (38.5)	72 (40.2)	47 (36.2)	
Male	190 (61.5)	107 (59.8)	83 (63.8)	
Years on hemodialysis, median (IQR)	4 (5)	4 (5)	5 (5)	
Infection with SARS-CoV-2 before booster-immunization, N	6	5	1	
Days between second and third vaccine dose (IQR)	188 (18)	175 (19)	189 (19)	
**Hemodialysis (HD) Patients after Fourth Vaccination**	**All HD Patients** **N = 182**	**4x** **BNT162b2** **N = 32** **(17.6%)**	**4x** **mRNA-1273** **N = 118** **(64.8%)**	**4x** **cross mRNA** **N = 32** **(17.6%)**
Age in years, median (IQR)	66 (23.5)	65 (18.3)	65 (17.3)	66 (19)
Sex, N (%)				
Female	68 (37.4)	14 (43.8)	41 (34.7)	13 (40.6)
Male	114 (62.6)	18 (56.3)	77 (65.3)	19 (59.4)
Years on hemodialysis, median (IQR)	4 (7)	4 (7)	4 (7)	5 (6)
Infection with SARS-CoV-2 before booster-immunization, N (%)	6	5	1	0
Days between second and fourth vaccine dose (IQR)	308 (25)	316.5 (18)	296 (21)	315.5 (4.5)

**Table 2:** SARS-CoV-2 anti-RBD IgG titers after third and fourth vaccination in hemodialysis (HD) patients. Vaccines used: mRNA-1273 (Moderna, CA, USA), BNT162b2 (BioNTech/Pfizer, Mainz, Germany/New York City, NY, USA).

Vaccination Status	SARS-CoV-2 Anti-RBD IgG, Median (IQR)
**Three doses (N = 309)**	2882.7 (3093.0)
**Four doses (all vaccines, N = 182)**	6923.5 (8661.2)
*Four doses BNT162b2 (N = 32)*	2741.7 (5327.7)
*Four doses mRNA-1273 (N = 118)*	8078.5 (7579.5)
*Four cross mRNA doses* *(N = 32)*	9789.3 (8876.3)

**Table 3:** Antibody cut-offs as a surrogate marker of neutralization against wild type (WT) and the omicron variant BA.1 after three and four vaccine doses (all vaccination schemes).

**Anti-RBD IgG Wild-Type Cut-Off** **(≥296 BAU/mL)**	**N, (%)**
After third dose	
<296 BAU/mL	37 (12.0)
≥296 BAU/mL	272 (88.0)
Total	309 (100.0)
After fourth dose	
<296 BAU/mL	9 (5.0)
≥296 BAU/mL	173 (95.0)
Total	182 (100.0)
**Anti-RBD IgG omicron cut-off** **(≥4159 BAU/mL)**	**N, (%)**
After third dose	
<4159 BAU/mL	214 (69.2)
≥4159 BAU/mL	95 (30.8)
Total	309 (100.0)
After fourth dose	
<4159 BAU/mL	65 (35.7)
≥4159 BAU/mL	117 (64.3)
Total	182 (100.0)

**Table 4:** Baseline characteristics of kidney transplant (KTX) recipients receiving third and fourth anti-SARS-CoV-2 vaccination. Vaccine used: mRNA-1273 (Moderna, CA, USA), BNT162b2 (BioNTech/Pfizer, Mainz, Germany/New York City, NY, USA), AZD1222 (AstraZeneca/University of Oxford, Cambridge/Oxford, UK) and Ad26.COV2.S (Johnson & Johnson, New Brunswick, NJ, USA).

Kidney Transplant Recipients (KTX)	N = 36
Age in years, median (IQR)	55 (20)
Sex, N (%)	
Female	14 (38.9)
Male	22 (61.1)
Years transplanted, median (IQR)	2 (4)
Underlying kidney disease	
ADPKD	14 (38.9)
Diabetic nephropathy	1 (2.8)
Glomerulonephritis	10 (27.8)
Hypertensive nephropathy	2 (5.6)
Other genetic nephropathy	2 (5.6)
Unknown	7 (19.4)
Donation procedure	
Living donation	26 (72.2)
*Living donation, ABO incompatible*	5 (13.9)
Deceased donation	10 (27.8)
Immunosuppressive therapy	
Tacrolimus, MMF/MPA, Prednisolone	28 (77.8)
Tacrolimus, Prednisolone	5 (13.9)
Tacrolimus, MMF/MPA	2 (5.6)
Tacrolimus, Azathioprine, Prednisolone	1 (2.8)
Basic immunization	
AZD1222	3 (8.3)
BNT162b2	26 (72.2)
mRNA-1273	7 (19.4)
Booster immunization	
AZD1222	2 (5.6)
BNT162b2	29 (80.6)
Ad26.COV2.S	1 (2.8)
mRNA-1273	4 (11.1)

**Table 5:** Cross-table of humoral and cellular immune responses after second and third vaccinations in a cohort of 36 kidney transplant (KTX) patients.

Cellular Immune Response after Second Vaccine Dose				n=
		Negative	Positive	
Anti-RBD IgG after second vaccine dose	negative	28	1	29
	positive	3	0	3
n=		31	1	32
		Cellular immune response after third vaccine dose		n=
		negative	positive	
Anti-RBD IgG after third vaccine dose	negative	18	1	19
	positive	9	1	10
n=		27	2	29

## Data Availability

Data supporting the reported results will be provided from the corresponding author upon reasonable request.

## Supplementary Materials

The following supporting information can be downloaded at: https://www.mdpi.com/article/10.3390/v14122646/s1, Supplementary Figure S1: (a) Passing Bablock fit for anti-RBD IgG and anti-S1 IgG; 95% CI for intercept: –155.7 to 17.51; 95% CI for slope 1.998 to 2.665; (b) passing Bablock fit for anti-S1 IgG and anti-trimeric spike protein IgG, 95% CI for intercept: −233.4 to 65.64; 95% CI for slope: 0.6146 to 0.8163; (c) passing Bablock fit for anti-RBD IgG and anti-trimeric spike protein IgG, 95% CI for intercept: −76.48 to 60.36; 95% CI for slope 0.2456 to 0.3382; Supplementary Figure S2: ROC analysis for anti-RBD IgG and WT neutralizing activity (≥1:250); Supplementary Table S1a: ROC analysis anti-RBD IgG and WT neutralizing activity (≥1:250); Supplementary Table S1b: ROC analysis for anti-RBD IgG and WT neutralizing activity (≥1:250); Supplementary Figure S3: ROC analysis for anti-RBD IgG and BA.1 neutralizing activity (≥1:10); Supplementary Table S2: ROC analysis anti-RBD IgG and BA.1 neutralizing activity (≥1:10); Supplementary Figure S4: ROC analysis for anti-S1 IgG, anti-trimeric spike protein IgG and WT neutralizing activity (≥1:250); Supplementary Table S3: ROC analysis anti-S1 IgG, anti-trimeric spike protein IgG and WT neutralizing activity (≥1:250), Supplementary Figure S5: ROC-Analysis for anti-S1 IgG, anti-trimeric spike protein IgG and BA.1 neutralizing activity (≥1:10), Supplementary Table S4: ROC analysis anti-S1/S1+S2-IgG and BA.1 neutralizing activity (≥1:250); Supplementary Table S5a: Number of patients reaching WT cut-off 296 BAU/mL after third vaccination; Supplementary Table S5b: Chi square test for reaching WT cut-off 296 BAU/mL after third vaccination; Supplementary Table S6a: Number of patients reaching BA.1 cut-off 4159 BAU/mL after third vaccination; Supplementary Table S6b: Chi-square test for reaching BA.1 cut-off 4159 BAU/mL after third vaccination; Supplementary Table S7a: Number of patients reaching WT cut-off 296 BAU/mL after fourth vaccination; Supplementary Table S7b: Chi square test for reaching WT cut-off 296 BAU/mL after fourth vaccination; Supplementary Table S8a: Number of patients reaching BA.1 cut-off 4159 BAU/mL after fourth vaccination; Supplementary Table S8b: Chi square test for reaching BA.1 cut-off 4159 BAU/mL after fourth vaccination; Supplementary Table S9: binary regression model for reaching BA.1 cut-off 4159 BAU/mL after 3 vaccinations; Supplementary Table S10: binary regression model for reaching BA.1 cut-off 4159 BAU/mL after 4 vaccinations; Supplementary Table S11: SARS-CoV-2 IgG and neutralizing serum activity after second and third vaccination in a cohort of 36 KTX patients.
