# Supplementary material for: Immune Response to Third and Fourth COVID-19 Vaccination in Hemodialysis Patients and Kidney Transplant Recipients
Source: Viruses. 2022 Nov 26;14(12):2646. doi: 10.3390/v14122646 (PMC9785871; doi:10.3390/v14122646)
Supplement: Supplementary file 1 [file viruses-14-02646-s001.zip › viruses-1971974-supplementary.pdf]

viruses
MDPI

Supplemental Material

Immune response to third and fourth COVID-19 vaccination in hemodialysis patients and kidney transplant recipients indicating lack of sufficient neutralizing activity against Omicron subtypes

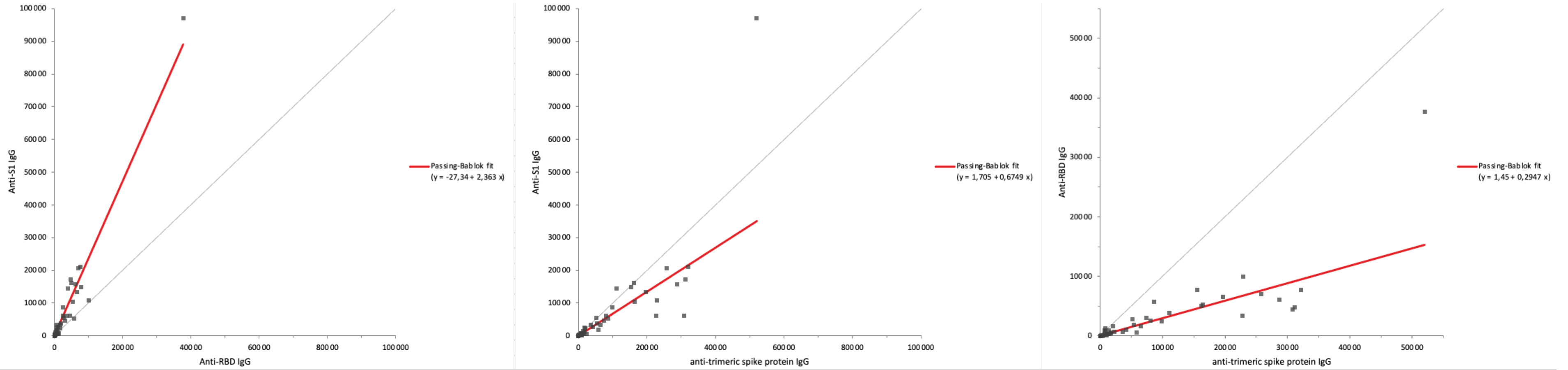

Supplemental Figure S1. (a) Passing Bablock fit for Anti-RBD IgG and Anti-S1 IgG; 95% CI for Intercept: -155.7 to 17.51; 95% CI for Slope 1.998 to 2.665. (b) Passing Bablock fit for Anti-S1 IgG and anti-trimeric spike protein IgG, 95% CI for Intercept: -233.4 to 65.64; 95% CI for Slope: 0.6146 to 0.8163. (c) Passing Bablock fit for Anti-RBD IgG and anti-trimeric spike protein IgG, 95% CI for Intercept: -76.48 to 60.36; 95% CI for Slope 0.2456 to 0.3382.

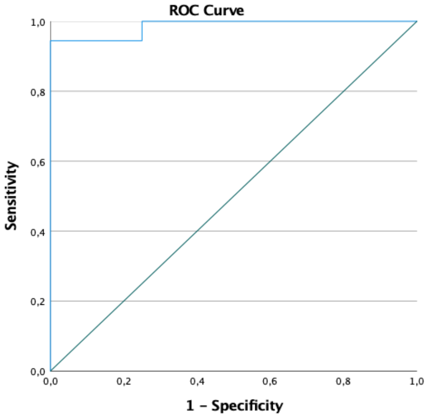

Supplemental Figure S2. ROC-Analysis for Anti-RBD IgG and WT neutralizing activity (≥1:250).

Supplemental Table S1a. ROC-Analysis of Anti-RBD IgG and WT neutralizing activity (≥1:250): Area under the Curve.

**Area Under the Curve**				
**Test Result Variable(s):**	**Anti-RBD IgG**			
Area	Std. Error[a]	Asymptotic Sig.[b]	Asymptotic 95% Confidence Interval	
			Lower Bound	Upper Bound
0.986	0.013	0.000	0.961	1.000
a. Under the nonparametric assumption				
b. Null hypothesis: true area = 0.5				

Supplemental Table S1b. ROC-Analysis of Anti-RBD IgG and WT neutralizing activity (≥1:250), cut-off with highest specificity and highest sensitivity was selected for further analysis.

Coordinates of the Curve Anti-RBD IgG

Positive if Greater Than or Equal To[a]	Sensitivity	1 - Specificity
0	1.000	1.000
0.15	1.000	0.833
1.45	1.000	0.750
2.85	1.000	0.667
12.05	1.000	0.583
22.50	1.000	0.500
37.00	1.000	0.417
76.00	1.000	0.333
124.00	1.000	0.250
166.50	0.944	0.250
239.00	0.944	0.083
296.00	0.944	0.000
303.50	0.917	0.000
324.00	0.889	0.000
405.00	0.861	0.000
513.50	0.833	0.000

562.00	0.806	0.000
600.00	0.778	0.000
639.00	0.750	0.000
717.00	0.722	0.000
835.50	0.694	0.000
919.00	0.667	0.000
965.50	0.639	0.000
1133.50	0.611	0.000
1458.00	0.583	0.000
1647.00	0.556	0.000
1742.50	0.528	0.000
2116.00	0.500	0.000
2466.50	0.472	0.000
2625.00	0.444	0.000
2877.50	0.417	0.000
3219.00	0.389	0.000
3632.50	0.361	0.000
4159.00	0.333	0.000
4615.50	0.306	0.000
4881.00	0.278	0.000
5114.50	0.250	0.000
5491.50	0.222	0.000
5931.50	0.194	0.000
6328.50	0.167	0.000
6764.00	0.139	0.000
7321.50	0.111	0.000
7702.00	0.083	0.000
8836.00	0.056	0.000
23811.50	0.028	0.000
37690.00	0.000	0.000

a. The smallest cutoff value is the minimum observed test value minus 1. and the largest cutoff value is the maximum observed test value plus 1. All the other cutoff values are the averages of two consecutive ordered observed test values.

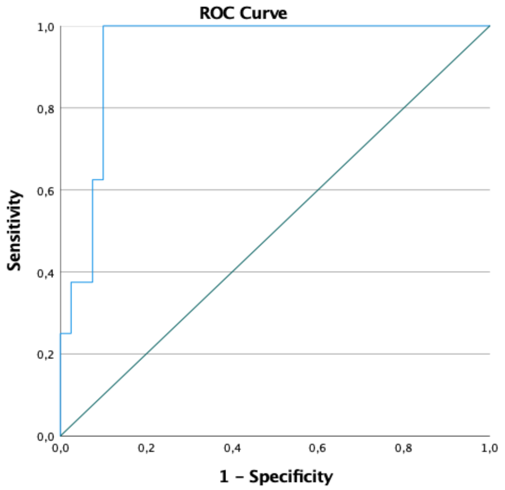

Supplemental Figure S3. ROC-Analysis of Anti-RBD IgG and BA.1 neutralizing activity (≥ 1:10).

Supplemental Table S2a. ROC-Analysis Anti-RBD IgG and BA.1 neutralizing activity (≥ 1:10): Area under the Curve.

Area Under the Curve				
**Test Result Variable(s):**	**Alinity (RBD)**			
Area	Std. Error[a]	Asymptotic Sig.[b]	Asymptotic 95% Confidence Interval	
			Lower Bound	Upper Bound
0.941	0.033	0.000	0.875	1.000

a. Under the nonparametric assumption
b. Null hypothesis: true area = 0.5

Supplemental Table S2b. ROC-Analysis for Anti-RBD IgG and BA.1 neutralizing activity (≥1:10): cut-off with specificity > 0.9 and highest sensitivity was selected for further analysis.

Coordinates of the Curve		
**Test Result Variable(s):**	**Alinity (RBD)**	
Positive if Greater Than or Equal To[a]	Sensitivity	1 - Specificity
0	1.000	1.000
0.15	1.000	0.950
1.45	1.000	0.925
2.85	1.000	0.900
12.05	1.000	0.875
22.50	1.000	0.850
37.00	1.000	0.825
76.00	1.000	0.800
124.00	1.000	0.775
166.50	1.000	0.725
239.00	1.000	0.675
296.00	1.000	0.650
303.50	1.000	0.625
324.00	1.000	0.600
405.00	1.000	0.575
513.50	1.000	0.550
562.00	1.000	0.525
600.00	1.000	0.500
639.00	1.000	0.475
717.00	1.000	0.450
835.50	1.000	0.425
919.00	1.000	0.400
965.50	1.000	0.375
1133.50	1.000	0.350
1458.00	1.000	0.325
1647.00	1.000	0.300
1742.50	1.000	0.275
2116.00	1.000	0.250
2466.50	1.000	0.225
2625.00	1.000	0.200
2877.50	1.000	0.175
3219.00	1.000	0.150
3632.50	1.000	0.125
4159.00	1.000	0.100

4615.50	0.875	0.100
4881.00	0.750	0.100
5114.50	0.625	0.100
5491.50	0.625	0.075
5931.50	0.500	0.075
6328.50	0.375	0.075
6764.00	0.375	0.050
7321.50	0.375	0.025
7702.00	0.250	0.025
8836.00	0.250	0.000
23811.50	0.125	0.000
37690.00	0.000	0.000

a. The smallest cutoff value is the minimum observed test value minus 1. and the largest cutoff value is the maximum observed test value plus 1. All the other cutoff values are the averages of two consecutive ordered observed test values.

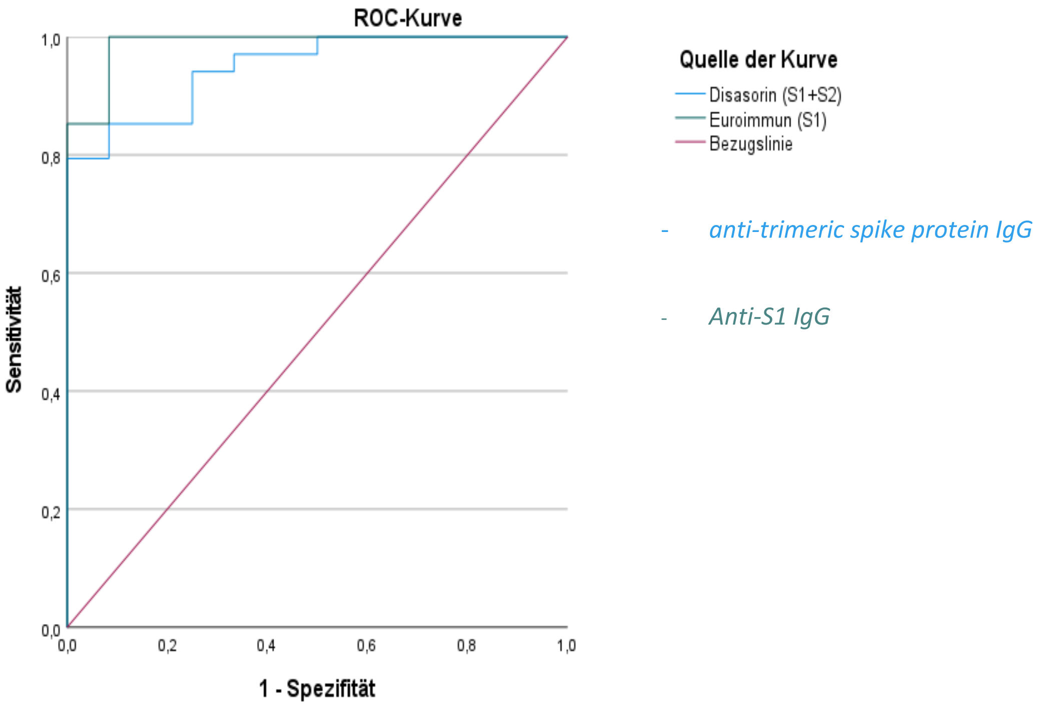

Supplemental Figure S4. ROC-Analysis of Anti-S1 IgG. anti-trimeric spike protein IgG and WT neutralizing activity (≥1:250).

Supplemental Table S3a. ROC-Analysis of Anti-S1 IgG, anti-trimeric spike protein IgG and WT neutralizing activity (≥1:250). Area under the Curve and Case processing summary

Area Under the Curve					
**Test Result Variable(s):**	**Area**	**Std. Error[a]**	**Asymptotic Sig.[b]**	**Asymptotic 95% Confidence Interval**	
				Lower Bound	Upper Bound

anti-trimeric spike protein IgG	0.949	0.030	0.000	0.890	1.000
Anti-S1 IgG	0.988	0.014	0.000	0.961	1.000

a. Under the nonparametric assumption; b. Null hypothesis: true area = 0.5.

Supplemental Table S3b. ROC-Analysis of Anti-S1, anti-trimeric spike protein IgG and WT neutralizing activity (≥1:250). Area under the Curve and Case processing summary, cut-off with specificity > 0.9 and highest sensitivity was selected for further analysis.

Coordinates of the Curve			
**Test Result Variable(s):**	**Positive if Greater Than or Equal To[a]**	**Sensitivity**	1 - Specificity
anti-trimeric spike protein IgG	0	1.000	1.000
	10.50	1.000	0.667
	30.50	1.000	0.583
	93.00	1.000	0.500
	250.50	0.971	0.500
	407.50	0.971	0.417
	570.00	0.971	0.333
	697.50	0.941	0.333
	716.50	0.941	0.250
	739.00	0.912	0.250
	786.50	0.882	0.250
	857.50	0.853	0.250
	961.00	0.853	0.167
	1100.00	0.853	0.083
	1250.00	0.824	0.083
	1395.00	0.794	0.083
	1595.00	0.794	0.000
	1850.00	0.735	0.000
	2130.00	0.706	0.000
	2920.00	0.676	0.000
	3840.00	0.647	0.000
	4640.00	0.618	0.000
	5280.00	0.588	0.000
	5625.00	0.559	0.000
	6175.00	0.529	0.000
	6950.00	0.500	0.000
	7740.00	0.471	0.000
	8360.00	0.441	0.000
	9240.00	0.412	0.000
	10480.00	0.382	0.000
	13300.00	0.353	0.000
	15840.00	0.324	0.000
	16330.00	0.294	0.000
	18070.00	0.265	0.000
	21240.00	0.235	0.000
	22850.00	0.206	0.000
	24350.00	0.176	0.000
	27275.00	0.147	0.000
	29775.00	0.118	0.000
	31000.00	0.088	0.000
	31675.00	0.059	0.000
	42075.00	0.029	0.000

	52001.00	0.000	0.000
Anti-S1 IgG	0	1.000	1.000
	1.705	1.000	0.833
	6.205	1.000	0.750
	19.50	1.000	0.667
	54.50	1.000	0.583
	84.50	1.000	0.500
	132.00	1.000	0.417
	216.00	1.000	0.333
	268.00	1.000	0.250
	282.00	1.000	0.167
	298.00	1.000	0.083
	405.50	0.971	0.083
	555.00	0.941	0.083
	613.50	0.912	0.083
	624.00	0.882	0.083
	639.00	0.853	0.083
	702.00	0.853	0.000
	773.00	0.824	0.000
	963.50	0.794	0.000
	1341.00	0.765	0.000
	1754.00	0.735	0.000
	2151.50	0.706	0.000
	2460.00	0.676	0.000
	2640.00	0.647	0.000
	3008.50	0.618	0.000
	3368.50	0.588	0.000
	3585.00	0.559	0.000
	4185.00	0.529	0.000
	4965.00	0.500	0.000
	5370.00	0.471	0.000
	5775.00	0.441	0.000
	6180.00	0.382	0.000
	7470.00	0.353	0.000
	9570.00	0.324	0.000
	10598.00	0.294	0.000
	12062.00	0.265	0.000
	13863.00	0.235	0.000
	14663.00	0.206	0.000
	15378.50	0.176	0.000
	15944.50	0.147	0.000
	16618.50	0.118	0.000
	18926.50	0.088	0.000
	20947.50	0.059	0.000
	59089.50	0.029	0.000
	97001.00	0.000	0.000

50

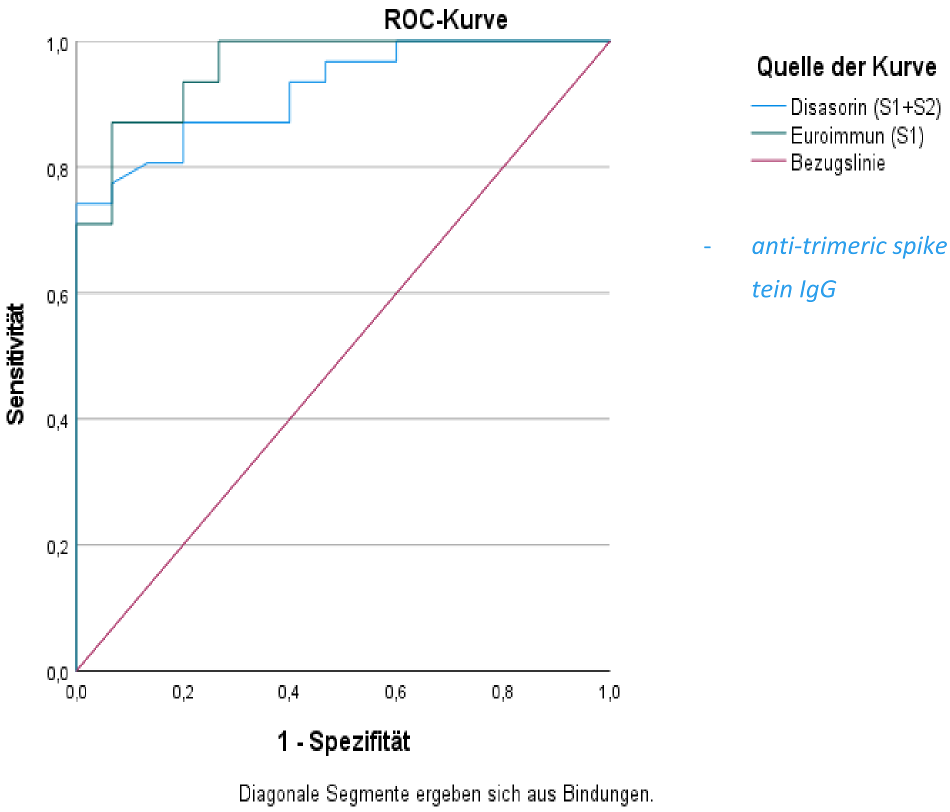

- *anti-trimeric spike protein IgG*

Supplemental Figure S5. ROC-Analysis of Anti-S1 IgG, anti-trimeric spike protein IgG and BA.1 neutralizing activity *(≥1:10).*

Supplemental Table S4a. ROC-Analysis of Anti-S1, anti-trimeric spike protein IgG and BA.1 neutralizing activity (≥1:250). Area under the Curve and Case processing summary.

Area under the curve					
**Test Result Variable(s):**	**Area**	**Std. Error[a]**	**Asymptotic Sig.[b]**	**Asymptotic 95% Confidence Interval**	
				Lower Bound	Upper Bound
anti-trimeric spike protein IgG	0.922	0.038	0.000	0.848	0.995
Anti-S1 IgG	0.959	0.026	0.000	0.908	1.000

a. Under the nonparametric assumption.
b. Null hypothesis: true area = 0.5.

Supplemental Table S4b. ROC-Analysis of Anti-S1 IgG, anti-trimeric spike protein IgG and BA.1 neutralizing activity (≥1:10). Cut-off with specfciity > 0.9 and highest sensitivity was selected for further analysis.

Coordinates of the Curve			
**Test Result Variable(s):**	**Positive if Greater Than or Equal To[a]**	**Sensitivity**	1 - Specificity
	0	1.000	1.000

anti-trimeric spike protein IgG	10.50	1.000	0.733
	30.50	1.000	0.667
	93.00	1.000	0.600
	250.50	0.968	0.600
	407.50	0.968	0.533
	570.00	0.968	0.467
	697.50	0.935	0.467
	716.50	0.935	0.400
	739.00	0.903	0.400
	786.50	0.871	0.400
	857.50	0.871	0.333
	961.00	0.871	0.267
	1100.00	0.871	0.200
	1250.00	0.839	0.200
	1395.00	0.806	0.200
	1595.00	0.806	0.133
	1850.00	0.774	0.067
	2130.00	0.742	0.067
	2920.00	0.742	0.000
	3840.00	0.710	0.000
	4640.00	0.677	0.000
	5280.00	0.645	0.000
	5625.00	0.613	0.000
	6175.00	0.581	0.000
	6950.00	0.548	0.000
	7740.00	0.516	0.000
	8360.00	0.484	0.000
	9240.00	0.452	0.000
	10480.00	0.419	0.000
	13300.00	0.387	0.000
	15840.00	0.355	0.000
	16330.00	0.323	0.000
	18070.00	0.290	0.000
	21240.00	0.258	0.000
	22850.00	0.226	0.000
	24350.00	0.194	0.000
	27275.00	0.161	0.000
	29775.00	0.129	0.000
	31000.00	0.097	0.000
	31675.00	0.065	0.000
	42075.00	0.032	0.000
	52001.00	0.000	0.000
Anti-S1 IgG	0	1.000	1.000
	1.705	1.000	0.867
	6.205	1.000	0.800
	19.50	1.000	0.733
	54.50	1.000	0.667
	84.50	1.000	0.600
	132.00	1.000	0.533
	216.00	1.000	0.467
	268.00	1.000	0.400
	282.00	1.000	0.333
	298.00	1.000	0.267
	405.50	0.968	0.267
	555.00	0.935	0.267
	613.50	0.935	0.200

624.00	0.903	0.200
639.00	0.871	0.200
702.00	0.871	0.133
773.00	0.871	0.067
963.50	0.839	0.067
1341.00	0.806	0.067
1754.00	0.774	0.067
2151.50	0.742	0.067
2460.00	0.710	0.067
2640.00	0.710	0.000
3008.50	0.677	0.000
3368.50	0.645	0.000
3585.00	0.613	0.000
4185.00	0.581	0.000
4965.00	0.548	0.000
5370.00	0.516	0.000
5775.00	0.484	0.000
6180.00	0.419	0.000
7470.00	0.387	0.000
9570.00	0.355	0.000
10598.00	0.323	0.000
12062.00	0.290	0.000
13863.00	0.258	0.000
14663.00	0.226	0.000
15378.50	0.194	0.000
15944.50	0.161	0.000
16618.50	0.129	0.000
18926.50	0.097	0.000
20947.50	0.065	0.000
59089.50	0.032	0.000
97001.00	0.000	0.000

a. The smallest cutoff value is the minimum observed test value minus 1. and the largest cutoff value is the maximum observed test value plus 1. All the other cutoff values are the averages of two consecutive ordered observed test values.

Supplemental Table S5a. Number of patients reaching WT cut-off 296 BAU/ml after third vaccination: No significant difference between BNT162b2 and mRNA-1273 group.

		Vaccines used		Total
		3x mRNA-1273	3x BNT162b2	
Ant-RBD IgG > 296 BAU/ml	No	9	18	27
	Yes	118	139	257
Total		127	157	284

Supplemental Table S5b. Chi-square Test for reaching WT cut-off 296 BAU/ml after third vaccination: No significant difference between BNT162b2 and mRNA-1273 group.

**Chi-Square Tests for reaching WT cut-off after third vaccination**

	Value	df	Asymptotic Significance (2-sided)	Exact Sig. (2-sided)	Exact Sig. (1-sided)
Pearson Chi-Square	1.564[a]	1	0.211		
Continuity Correction[b]	1.097	1	0.295		
Likelihood Ratio	1.601	1	0.206		
Fisher's Exact Test				0.229	0.147
Linear-by-Linear Association	1.559	1	0.212		
N of Valid Cases	309				

a. 0 cells (.0%) have expected count less than 5. The minimum expected count is 12.07; b. Computed only for a 2x2 table.

Supplemental Table S6a. Number of patients reaching BA.1 cut-off 4159 BAU/ml after third vaccination: significant difference between BNT162b2 and mRNA-1273 (p=0.002).

		Vaccines used		Total
		3x mRNA-1273	3x BNT162b2	
RBD IgG > 4159 BAU/ml	no	75	139	214
	yes	55	40	95
Total		130	179	309

Supplemental Table S6b. Chi-square Test for reaching BA.1 cut-off 4159 BAU/ml after third vaccination: significant difference between BNT162b2 and mRNA-1273 (p=0.002).

Chi-Square Tests for reaching BA.1 cut-off after third vaccination				
		df	Asymptotic Significance (2-sided)	Exact Sig. (2-sided)
Pearson Chi-Square		1	0.002	
Continuity Correction[b]		1	0.002	
Likelihood Ratio		1	0.002	
Fisher's Exact Test				0.002
Linear-by-Linear Association		1	0.002	
N of Valid Cases	309			

Supplemental Table S7a. number of patients reaching WT cut-off 296 BAU/ml after fourth vaccination: no significant difference (p=0.49).

		RBD IgG > 296 BAU/ml		Total
		no	yes	
Vacination scheme	4x MRNA-1273	5	113	118
	4x BNT162b2	2	30	32
	Cross mRNA Vaccination	2	30	32
Total		9	173	182

Supplemental Table S7b. Chi-square Test for reaching WT cut-off 296 BAU/ml after fourth vaccination: no significant difference (p=0.49) between 4x BNT162b2 and 4x mRNA-1273.

Chi-Square Tests for reaching WT cut-off after fourth vaccination

		df	Asymptotic Significance (2-sided)
Pearson Chi-Square		2	0.836
Likelihood Ratio		2	0.841
Linear-by-Linear Association		1	0.582
N of Valid Cases	182		

Supplemental Table S8a. Number of patients reaching BA.1 cut off 4159 BAU/ml after fourth vaccination: more often after 4x mRNA-1273 than 4X BNT162b2 (p > 0.001).

		RBD IgG > 4159 BAU/ml		Total
		no	yes	
Vacination scheme	4x mRNA-1273	32	86	118
	4x BNT162b2	22	10	32
Total		54	96	150

Supplemental Table S8b. Chi-square Test for reaching BA.1 cut off 4159 BAU/ml after fourth vaccination: more often after 4x MRNA-1273 than 4X BNT162b2 (p > 0.001).

Chi-Square Tests for reaching BA.1 cut-off after fourth vaccination

		df	Asymptotic Significance (2-sided)	Exact Sig. (2-sided)	Exact Sig. (1-sided)
Pearson Chi-Square		1	0.000		
Continuity Correction[b]		1	0.000		
Likelihood Ratio		1	0.000		
Fisher's Exact Test				0.000	0.000
Linear-by-Linear Association		1	0.000		
N of Valid Cases	182				

a. 0 cells (0.0%) have expected count less than 5. The minimum expected count is 11.52; b. Computed only for a 2x2 table.

Supplemental Table S9. Binary regression model for reaching BA.1 cut-off 4159 BAU/ml after 3 vaccinations: after 3x MRNA-1273 it is more likely to reach > 4159 BAU/ml (p=0.001). Adjusted for age, sex, years of dialysis therapy, previous infection with SARS-COV2 before booster immunisation, days between basic and booster immunisation and days between vaccination and blood sample.

Binary regression model for reaching BA.1 cut-off after third vaccination

Step	-2 Log likelihood	Cox & Snell R Square	Nagelkerke R Square
1	308.059[a]	0.179	0.238

a. Estimation terminated at iteration number 20 because maximum iterations has been reached. Final solution cannot be found.

Classification Table[a]

Observed	Predicted		
	CutOff_BA.1_3Vacc		Percentage Correct
	.00	1.00	

Step 1	RBD IgG > 4159 BAU/ml	no	166	7	96.0		
		yes	75	11	12.8		
	Overall Percentage				68.3		
a. The cut value is .500							
**Variables in the Equation**							
		B	S.E.	Wald	df	Sig.	Exp(B)
Step 1[a]	Age (years)	-0.006	0.010	0.384	1	0.535	0.994
	Sex	0.062	0.266	0.055	1	0.815	1.064
	Years HD	-0.041	0.029	2.028	1	0.154	0.960
	Infection with SARS-COV2 (before booster immunisation)	-20.099	15137.968	0.000	1	0.999	0.000
	Vaccine used	-1.238	0.374	10.942	1	0.001	0.290
	Days between basic- and booster immunization	0.011	0.005	4.365	1	0.037	1.011
	Days between last Vaccination and blood sample	-0.008	0.011	0.524	1	0.469	0.992

Supplemental Table S10. Binary regression model for reaching BA.1 cut-off 4159 BAU/ml after 4 vaccinations: after 4x mRNA-1273 it is not more likely to reach > 4159 BAU/ml (p=0.178), adjusted for age, sex, years of dialysis therapy, days between basic and booster immunisation.

Binary regression model for reaching BA.1 cut-off after fourth vaccination

**Step**	**-2 Log likelihood**	**Cox & Snell R Square**	Nagelkerke R Square
1	139.828[a]	0.190	0.254

a. Estimation terminated at iteration number 4 because parameter estimates changed by less than .001.

Classification Table[a]

Observed			Predicted		
			RBD IgG > 4159 BAU/ml		% Correct
			no	yes	
Step 1	RBD IgG > 4159 BAU/ml	no	3	31	8.8
		yes	0	85	100.0
	Overall Percentage				73.9

a. The cut value is .500

Variables in the Equation

		B	S.E.	Wald	df	Sig.	Exp(B)
Step 1[a]	Vacination scheme	-2.210	1.324	2.787	1	0.095	0.110
	Age (years)	0.005	0.014	0.141	1	0.708	1.005
	Sex	-0.091	0.427	0.046	1	0.831	0.913
	Years HD	-0.003	0.042	0.005	1	0.945	0.997
	Days between basic - and booster immunization	0.017	0.011	2.673	1	0.102	1.017

a. Variable(s) entered on step 1: Vaccination scheme. Age (years). Sex. Years HD. Days Ground Booster.

Supplemental Table S11. SARS-COV2 IgG and neutralizing serum activity after second and third vaccination in a cohort of 36 KTx patients.

**Anti-RBD IgG BAU/ml after 2. Vaccination n=3**	Patient 1	82.70
	Patient 2	18.3
	Patient 3	130.4
**Anti-RBD IgG BAU/ml after 3. Vaccination N=10**	Median	134.4
	Interquartile Range	218.4
neutralizing serum acivity WT (>/= 1:10)		Anti-RBD IgG
after 2. Vaccination	0	
after 3. Vaccination	2 patients	828,1 BAU/ml
		358,6 BAU/ml
